# Comparison of Approaches for Stroke Prophylaxis in Patients with Non-Valvular Atrial Fibrillation: Network Meta-Analyses of Randomized Controlled Trials

**DOI:** 10.1371/journal.pone.0163608

**Published:** 2016-10-05

**Authors:** Navkaranbir S. Bajaj, Rajat Kalra, Nirav Patel, Taimoor Hashim, Hemant Godara, Sameer Ather, Garima Arora, Tilak Pasala, Thomas T. Whitfield, David C. McGiffin, Mustafa I. Ahmed, Steven G. Lloyd, Nita A. Limdi, Pankaj Arora

**Affiliations:** 1 Division of Cardiovascular Medicine, Brigham and Women’s Hospital and Harvard Medical School, Boston, MA, United States of America; 2 Division of Cardiovascular Disease, University of Alabama at Birmingham, Birmingham, AL, United States of America; 3 Cardiovascular Division, Department of Medicine, University of Minnesota, Minneapolis, MN, United States of America; 4 Department of Medicine, University of Alabama at Birmingham, Birmingham, AL, United States of America; 5 Division of Cardiovascular Medicine, University of Utah School of Medicine, Salt Lake City, UT, United States of America; 6 Division of Cardiac Surgery, Department of Surgery, Alfred Hospital and Monash University, Melbourne, Australia; 7 Division of Cardiology, Baptist Princeton, Birmingham, Alabama, United States of America; 8 Department of Neurology, University of Alabama at Birmingham, Birmingham, Alabama, United States of America; Universita degli Studi di Perugia, ITALY

## Abstract

**Background:**

Multiple novel oral anticoagulants and left atrial appendage closure devices (WATCHMAN) have been tested against dose-adjusted vitamin K antagonists in randomized controlled trials for stroke prophylaxis in non-valvular atrial fibrillation. No direct comparisons of these strategies are available from randomized controlled trials. We conducted the current analyses by combining efficacy and safety characteristics of all FDA approved stroke prophylaxis treatment strategies for patients with non-valvular atrial fibrillation.

**Materials and Methods:**

We searched SCOPUS from 1945 till October 2015 for randomized controlled trials comparing these strategies and reporting efficacy and safety outcomes. Six randomized controlled trials were identified and included in the final analyses and review. We followed PRISMA guidelines for network meta-analyses while reporting the current analyses. We collected data on ischemic stroke, major bleeding, and the composite primary safety endpoint as defined by various randomized controlled trials. Network meta-analyses were conducted using consistency and inconsistency models for efficacy and safety outcomes. Surface under the cumulative ranking curve were then utilized to cluster rank these treatments for safety and efficacy.

**Results:**

Six randomized controlled trials with 59,627 patients comparing six treatment strategies were eligible for the analyses. All prophylaxis strategies had comparable rates of ischemic stroke. Apixaban was associated with the least number of primary safety endpoint events as compared with all other treatments. In the cluster analyses assessing safety and efficacy, apixaban, edoxaban and dabigatran ranked best followed by vitamin K antagonists and rivaroxaban, whereas the WATCHMAN left atrial appendage closure device ranked last.

**Conclusions:**

Dose-adjusted vitamin K antagonists, novel oral anticoagulants, and the WATCHMAN left atrial appendage closure devices are equally efficacious for ischemic stroke prevention but these treatments have different safety profiles. More randomized controlled trials are needed to directly compare these strategies.

## Introduction

Atrial fibrillation (AF) is the most common cardiac arrhythmia with increasing incidence and prevalence in the community [1]. Atrial fibrillation is a major risk factor for stroke, cardiovascular morbidity, and mortality and contributes significantly to healthcare burden [2, 3].

Oral dose-adjusted vitamin K antagonists have been the mainstay of treatment for stroke prophylaxis in patients with non-valvular atrial fibrillation. Multiple novel oral anticoagulants have been tested against dose-adjusted vitamin K antagonists in large randomized controlled trials in a non-inferiority fashion and are currently approved for clinical practice by the United States Food and Drug Administration (FDA) [4–7]. The WATCHMAN left atrial appendage closure device was recently approved by the FDA to reduce the risk of thromboembolism in patients with non-valvular atrial fibrillation based on the results of two randomized controlled trials comparing WATCHMAN left atrial appendage closure devices with dose-adjusted vitamin K antagonists [8–10]. No direct comparisons are available from randomized controlled trials testing the different novel oral anticoagulants against each other or left atrial appendage closure devices to novel oral anticoagulants. However, multiple meta-analyses have been previously published comparing novel oral anticoagulants against dose-adjusted vitamin K antagonists for either efficacy or safety outcomes [11–16]. These analyses are limited by inclusion of studies with non-FDA approved doses, pooling of multiple novel oral anticoagulants and different doses as one group, lack of safety outcomes, and non-inclusion of left atrial appendage closure devices [WATCHMAN (Boston Scientific, Marlborough, MA, U.S.A.)].

The advent of multiple treatment pathways has presented dilemmas for the clinicians with the choice of strategy (newer pharmacological, i.e., novel oral anticoagulants versus anatomical, i.e., WATCHMAN left atrial appendage closure devices) and choice of agent (novel oral anticoagulants versus dose-adjusted vitamin K antagonists) for stroke prophylaxis in non-valvular atrial fibrillation patients. As direct evidence from randomized controlled trials is lacking, indirect comparisons using systematic network meta-analyses can provide useful complementary information that may be less biased than the direct evidence [17, 18]. In addition, a scenario like this provides a unique opportunity to undertake a “trade-off” analysis, which allows for the comparison of both the safety and efficacy profiles of these strategies. With the aforementioned objective, we conducted network meta-analyses to compare the efficacy and safety of all FDA approved treatment strategies of stroke prevention in atrial fibrillation by synthesizing evidence from available randomized controlled trials comparing novel oral anticoagulants, dose-adjusted vitamin K antagonists, and left atrial appendage closure devices.

## Methods

We report this systematic review and analyses in accordance with Preferred Reporting Items for Systematic Reviews and Meta-Analyses (PRISMA) extension statement for network meta-analyses [19, 20]. All phase III randomized controlled trials published between 1966 and October 2015 that compared dose-adjusted vitamin K antagonists with newer treatment strategies, including novel oral anticoagulants and WATCHMAN devices, were identified. All randomized controlled trials reporting data on FDA approved doses of novel oral anticoagulants in the United States and those that reported ischemic stroke in patients with non-valvular atrial fibrillation (regardless of risk profiles and comorbidities) were included. The primary efficacy outcome of interest for our network meta-analyses was ischemic stroke during follow-up. Ischemic stroke was defined as persistent clinical neurologic deficit of vascular origin persisting beyond 24 hours. We used the definition of stroke outlined by the individual trial’s authors rather than creating our own. In addition, we also extracted data for major bleeding and the primary safety endpoint. Major bleeding was defined as per the International Society of Thrombosis and Hemostasis [21]. Data from FDA documents for the WATCHMAN trials were utilized to calculate the major bleeding events. The primary safety endpoint was defined as a composite of major bleeding and clinically relevant non-major bleeding in randomized controlled trials of novel oral anticoagulants and a composite of major bleeding and any device-related complications in the WATCHMAN left atrial appendage closure device randomized controlled trials. The detailed definitions of these endpoints are presented in **S1 Table**. Studies comparing dose-adjusted vitamin K antagonists to aspirin/clopidogrel or any combination thereof were excluded. Studies comparing betrixaban, ximelagatran, idraparinaux, triflusal and indobufen to dose-adjusted vitamin K antagonists were also excluded, as these treatments are not FDA approved in the United States.

Multiple authors (P.A., T.H. and T.T.W.) independently performed an electronic literature search in SCOPUS using a predefined keywords list. A fourth investigator (N.S.B.) verified the search results **(S1 Section)**. All English language human studies published in full-text or abstract forms were eligible for inclusion. After initial abstract review, all potentially relevant studies pertaining to dose-adjusted vitamin K antagonists, left atrial appendage closure devices, and novel oral anticoagulants were identified and the full-text publications were retrieved for detailed evaluation. When more than one publication from the same patient population existed, the study with the most complete data set was included for the meta-analyses. Furthermore, reference lists of potentially relevant reports and reviews were screened to identify other eligible studies. We also searched clinicaltrials.gov and FDA websites for public summary data reported for the included randomized controlled trials. Data quality of the included studies was assessed by two independent investigators (R.K. and T.H.) using the JADAD Score for randomized controlled trials, which examines the methods of randomization, double blinding, and the reporting of dropouts [22]. The GRADE guidelines were used to assess the quality of direct and indirect comparisons [23]. All discrepancies in data extraction were resolved by mutual consensus. The consensus process to resolve disagreements required investigators to discuss the decisions with mandatory recognition of errors from one of the reviewers.

Statistical analyses were performed using Stata 14.0 MP (Stata Corp., College Station, Texas) and study results are reported in accordance with the PRISMA extension statement for reporting of network meta-analyses [24]. Network meta-analyses were conducted using multivariate random effects metaregression to estimate direct and indirect treatment comparisons by fitting a consistency model [25, 26]. These results were graphically presented using interval plots and network forest plots as described by White et al, where the estimates from within the randomized controlled trial and overall network meta-analysis estimates are plotted on a Log scale [26]. The surface under rankogram curve (SUCRA) statistic was estimated from the consistency modeling and rankograms were plotted for individual treatments for efficacy and safety outcomes [27]. We subsequently performed cluster analysis, an exploratory data mining technique for grouping objects, based on their features so that the degree of association is high between members of the same group and low between members of different groups [28]. The treatment strategies were cluster ranked based on SUCRA statistic values estimated from the above analyses to identify best treatment in terms of efficacy and safety, which we termed as “the trade-off” [27].

## Results

**Fig 1:**
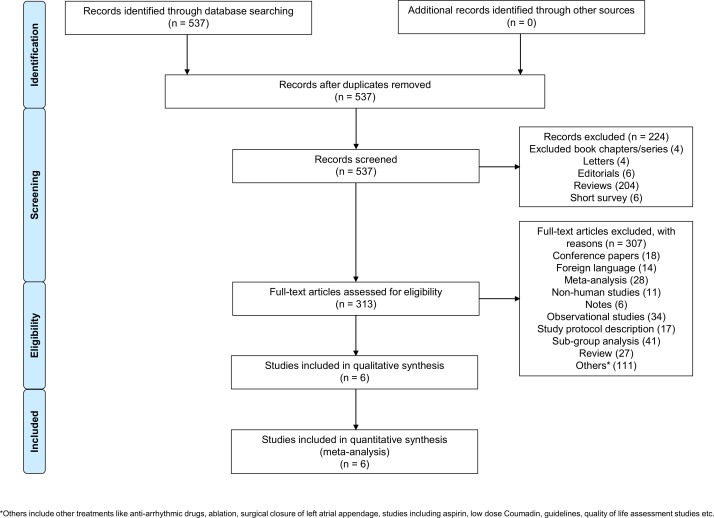
Flow diagram for study selection in accordance with PRISMA guidelines.

**Fig 2:**
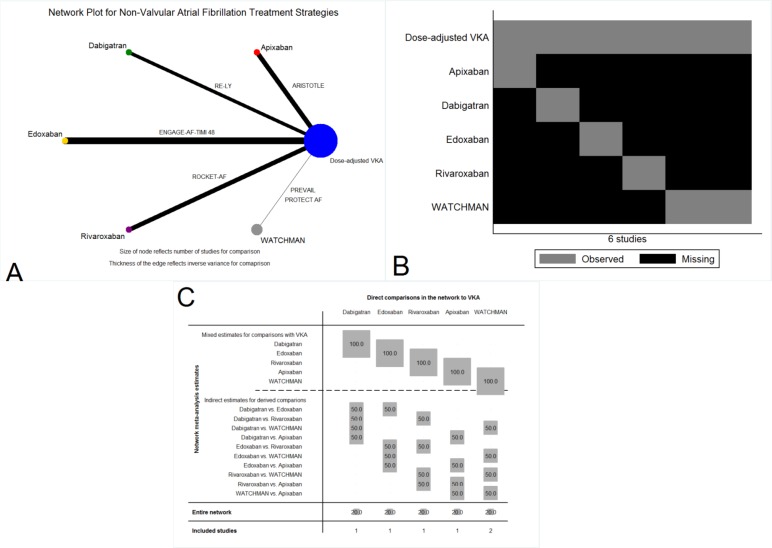
*Panel A*: Network plot for the stroke prophylaxis network, *Panel B*: Network pattern for the stroke prophylaxis network, *Panel C*: Contribution plot for the stroke prophylaxis network. The size of each square is proportional to the weight attached to each direct summary effect (horizontal axis) for the estimation of each network summary effects (vertical axis). The numbers re-express the weights as percentages. *VKA = Dose-adjusted vitamin K antagonists*.

We included six eligible randomized controlled trials [4–7, 9, 10] **(Fig 1)** with 59,627 patients in the analyses **(Table 1** and **Fig 2, Panel A)**. The results of this network meta-analysis are presented in accordance with PRISMA extension statement for reporting of network meta-analyses **(S2 Table)**. We compared FDA approved doses of novel oral anticoagulants (apixaban 5 mg twice daily, dabigatran 150 mg twice daily, edoxaban 60 mg daily and rivaroxaban 20 mg daily), WATCHMAN left atrial appendage closure device, and dose-adjusted vitamin K antagonists to one another. Dose-adjusted vitamin K antagonist therapy was the common comparator in all studies. The WATCHMAN left atrial appendage closure device was compared to dose-adjusted vitamin K antagonists in two randomized controlled trials whereas the remaining treatment strategies were each compared to dose-adjusted vitamin K antagonists in a single randomized controlled trial. The number of patients in the WATCHMAN left atrial appendage closure device trials was the lowest in comparison with novel oral anticoagulants and dose-adjusted vitamin K antagonists. Hence the WATCHMAN left atrial appendage closure device contributed the least to the network of evidence **(Fig 2)**. The mean/median follow-up period of the trials was 1.5–2.8 years. Based on the JADAD scale, all included studies were categorized as good to excellent **(Table 1)**. The direct and indirect comparisons among various strategies varied from high quality (++++) to low quality (++) per the GRADE guidelines **(S6 Table)**. The mean CHADS2 score was >3 in ROCKET-AF whereas the score varied between 2–3 in other studies **(Table 1)**. The baseline antiplatelet therapy and percentage of time in therapeutic range in the dose-adjusted vitamin K antagonist arm among studies is presented in **Table 2**. All studies reported ischemic stroke, major bleeding, and the primary safety endpoint.

**Table 1:** Baseline Characteristics of Included Trials.

Study Name/First Author[Reference]	Publication Year	N, patients	Age Median/Mean, Years	N, Gender (Male)	N, Hypertension	N, Diabetes	N, Heart Failure	N, Stroke	CHADS2 (Mean ± 2SD)	Median/ Mean Follow-up in Years
**VKA arm versus Rivaroxaban**										
ROCKET AF/Patel[4]	2011	7133/7131	73/73	4301/4300	6474/6436	2817/2878	4441/4467	3895/3916	3.46±0.95/3.48±0.94	1.9yr
**VKA arm versus Apixaban**										
ARISTOTLE/Granger[5]	2011	9081/9120	70/70	5899/5886	7954/7962	2263/2284	3216/3235	1790/1748	2.1±1.1/2.1±1.1	1.8yr
**VKA arm versus Dabigatran**										
RE-LY/Connolly[6]	2009	6022/6076	72/72	3809/3840	4750/4795	1410/1402	1922/1934	1195/1233	2.1±1.1/2.2±1.1	2.0yr
**VKA arm versus Edoxaban**										
ENGAGE AF-TIMI 48/Giugliano[7]	2013	7036/7034	72/72	4395/4304	6588/6575	2521/2544	4048/3979	1991/2006	2.8±1.0/2.8±1.0	2.8yr
**VKA arm versus WATCHMAN**										
PROTECT AF/Reddy[9]	2009	244/463	73/72	171/326	220/413	72/113	66/124	49/82	*2.3/2.2	4.0yr
PREVAIL/Holmes[10]	2014	138/269	75/74	103/182	134/238	41/91	32/63	39/74	2.6±1.0/2.6±1.0	2.2yr

Legend: VKA = Dose-adjusted Vitamin K antagonists.

* = Mean CHADS2 was reported as categorical variable and mean was calculated by using standard formulae.

JADAD Score: 0–2: poor quality, 3–4: good quality, 5: excellent quality.

**Table 2:** Baseline Medication Use of Included Trials.

Study Name/First Author [Reference]	Comparison	N, VKA/ Treatment	N, Aspirin Use at Baseline	N, VKA at Baseline Prior to Randomization	N, Thienopyridine Use at Baseline	TTR in VKA Arm (mean/median %)
ROCKET AF/ Patel[4]	VKA versus rivaroxaban	7133/7131	2619/2586	4461/4443	-	55
ARISTOTLE/ Granger[5]	VKA versus apixaban	9081/9120	2773/2859	5193/5208	168/ 170	62
RE-LY/ Connolly[6]	VKA versus dabigatran	6022/6076	2442/2352	2929/3049	-	64
ENGAGE AF-TIMI 48/ Giugliano[7]	VKA versus edoxaban	7036/7034	2070/2092	4138/4140	164/174	68
PROTECT AF/ Reddy[9]	VKA versus WATCHMAN	244/463	NR/463*	145/254	-*	66
PREVAIL/ Holmes[10]	VKA versus WATCHMAN	138/269	NR	NR	-*	68

Legend:— = Not reported, N = Number, TTR = Time in therapeutic range, VKA = Dose-adjusted Vitamin K antagonists.

* = All patients in the device group received VKA up to 45 days. After that, if there was no evidence of residual left atrial appendage or device peri-leak on transesophageal echocardiography, then the patients were switched to aspirin and clopidogrel for six months after which only aspirin 325mg daily dose was continued for the remainder of the follow-up.

**Fig 3:**
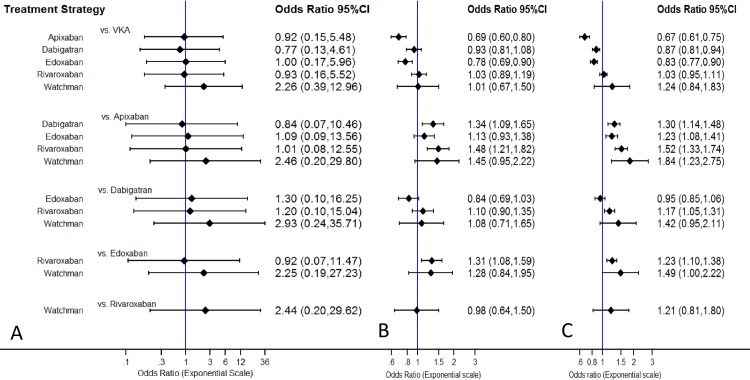
Interval plot for ischemic stroke in the stroke prophylaxis network. *Panel A*: Ischemic Stroke, *Panel B*: Major Bleeding, *Panel C*: Primary Safety Endpoint. The solid black lines represent the confidence intervals for summary odds ratios for each comparison. The blue line is the line of no effect (odds ratio equal to 1). *VKA = Dose-adjusted vitamin K antagonists*.

**Fig 4:**
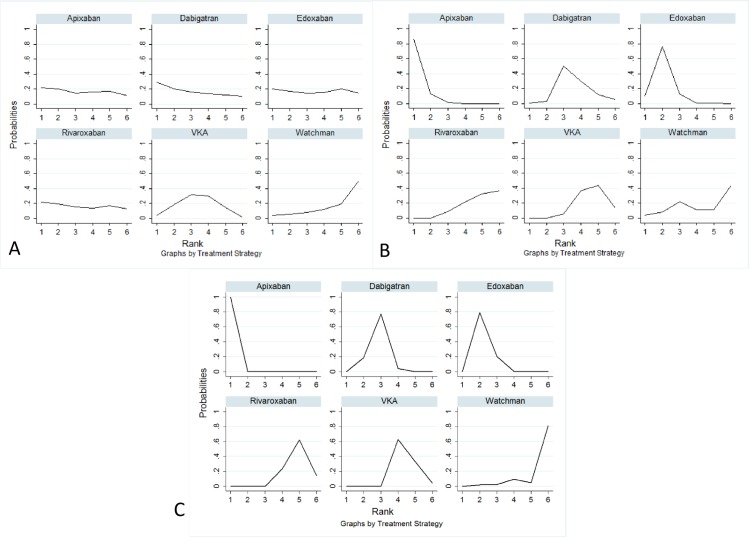
Surface under the cumulative ranking curve plots for all treatments in the stroke prophylaxis network. *Panel A*: Rankogram for Ischemic Stroke, *Panel B*: Rankogram for Major Bleeding, *Panel C*: Rankogram for Primary Safety Endpoint. *VKA = Dose-adjusted vitamin K antagonists*.

The odds ratios for ischemic stroke rate comparisons for all possible treatment strategies are presented in **S3 Table**. All strategies were equivalent in terms of ischemic stroke rates **(Fig 3, Panel A)**. We plotted rankograms using SUCRA statistics estimated from consistency modeling **(Fig 4)**. Dabigatran 150 mg twice daily had the highest probability (~29%) of being the best strategy for ischemic stroke reduction, whereas dose-adjusted vitamin K antagonists and the WATCHMAN left atrial appendage closure device (~4%) had the lowest probabilities of being the best strategy for ischemic stroke reduction.

The odds ratios for major bleeding comparisons for all possible treatment strategies (novel oral anticoagulants, WATCHMAN left atrial appendage closure device, and dose-adjusted vitamin K antagonists) are presented in **S4 Table**. Apixaban had significantly lower rates of major bleeding events than dose-adjusted vitamin K antagonists, dabigatran, and rivaroxaban. Edoxaban had significantly lower major bleeding events than dose-adjusted vitamin K antagonists and rivaroxaban. Apixaban, edoxaban, and the WATCHMAN left atrial appendage closure device had a similar rate of major bleeding events **(Fig 3, Panel B)**. Apixaban 5 mg twice daily had the highest probability (~86%) whereas dose-adjusted vitamin K antagonists and rivaroxaban had the lowest probability (~0%) of being the best strategy for the lowest major bleeding events **(Fig 4)**.

The odds ratios to compare the incidence of the composite primary safety endpoint between all possible treatment strategies are presented in **S5 Table**. Apixaban 5 mg twice daily appeared superior to all strategies with the lowest odds of primary safety events **(Fig 3, Panel C)**. Apixaban 5 mg twice daily had the highest probability (~99.8%) of being the best strategy for the lowest primary safety endpoint **(Fig 4)**.

**Fig 5:**
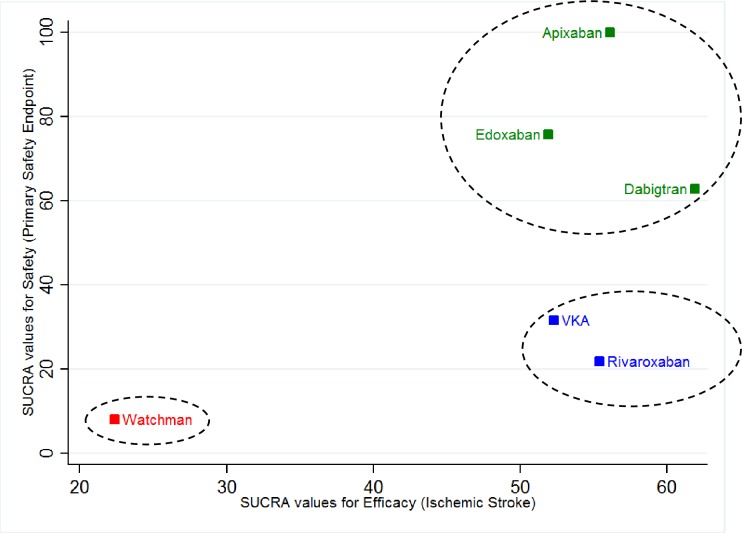
Clustered ranking plot of the stroke prophylaxis network. This plot is based on cluster analysis of SUCRA values for efficacy and safety. Each color represents a group of treatments that belong to the same cluster. Treatments lying in the upper right corner are more effective and safe than the other treatments. *VKA = Dose-adjusted vitamin K antagonists*.

Cluster ranking based on SUCRA statistic values for ischemic stroke and primary safety endpoint were used to provide the hierarchical clusters for the six active treatments. Cluster ranking revealed that the cluster containing apixaban, edoxaban, and dabigatran was the best ranked cluster, followed by the cluster containing dose-adjusted vitamin K antagonists and rivaroxaban, whereas the cluster containing WATCHMAN left atrial appendage closure device was ranked the worst **(Fig 5)**.

The direct evidence estimates for the vitamin K antagonists versus novel anticoagulants for all outcomes are quite precise. This is likely due to the large numbers of patients in the trials comparing vitamin K antagonists to novel oral anticoagulants **(S1–S3 Figs)**. However, the WATCHMAN to vitamin K antagonists comparison direct evidence estimates are obtained from smaller and heterogeneous trials (**S1–S3 Figs**). This leads to imprecision and to the downgrading of GRADE evidence of these estimates from 4 to 3 **(S6 Table)**.

## Discussion

Our systematic review and network meta-analyses highlight the diverse risk profiles and significant differences in the composite primary safety endpoint and major bleeding associated with various stroke prophylaxis strategies for patients with non-valvular atrial fibrillation. Overall, all treatment strategies were similar in terms of ischemic stroke rates, which was the primary efficacy outcome of our analysis. Apixaban had lower odds of major bleeding events than dose-adjusted vitamin K antagonists, dabigatran, and rivaroxaban. Moreover, apixaban was the best among all available treatments for reducing the overall primary composite safety endpoint, which included rates of major bleeding. Furthermore, in our systematic network meta-analyses, we also report cluster analyses in order to establish a hierarchy for the most efficacious and safe treatment in non-valvular atrial fibrillation patients. These analyses revealed that the cluster containing apixaban, edoxaban, and dabigatran was the best-ranked cluster, followed by the dose-adjusted vitamin K antagonists and rivaroxaban cluster. The cluster containing the WATCHMAN left atrial appendage closure device was ranked the lowest.

The findings of our analysis may have several explanations. We observed similar rates of the primary efficacy outcome, ischemic stroke rate, among novel oral anticoagulants and anatomical (WATCHMAN left atrial appendage closure devices) treatment strategies when compared with dose-adjusted vitamin K antagonists and against each other **(Fig 3, Panel A)**. This finding may be explained by the fact that interrupting the coagulation cascade by pharmacological agents or placing the WATCHMAN left atrial appendage closure device would be equally efficacious in preventing thrombus formation and subsequent thrombus embolization.

The odds of major bleeding were significantly lower with apixaban and edoxaban, as compared to dose-adjusted vitamin K antagonists **(Fig 3, Panel B)**. This can be partly explained by the fact that apixaban is a Factor Xa inhibitor with bleeding rates that have been shown to be comparable to aspirin in non-valvular atrial fibrillation patients [29]. Although, rivaroxaban is also a factor Xa inhibitor, we found no differences in the major bleeding rates when compared to dose-adjusted vitamin K antagonists. One possible explanation could be the fact that the ROCKET-AF trial enrolled patients with the mean CHADS2 score >3, unlike other novel oral anticoagulant trials **(Table 1)** [4]. Quantitatively, the CHADS2 score has been significantly correlated with higher bleeding risk in population studies [30].

Apixaban, dabigatran, and edoxaban had a significantly favorable primary safety endpoint profile (which was defined as a composite of major and clinically relevant non-major bleeding for the novel anticoagulants) when compared with dose-adjusted vitamin K antagonists. Apixaban was superior to all available treatment options including the anatomical strategy (WATCHMAN left atrial appendage closure device) where the primary safety endpoint was a composite of major bleeding and device-related complications **(Fig 3, Panel C)**. The lower incidence of the primary safety endpoint in the anatomical strategy (WATCHMAN left atrial appendage closure device) was primarily driven by the higher rate of peri-procedural complication rates in the WATCHMAN left atrial appendage closure device randomized controlled trials [9, 10]. Major bleeding rates for the anatomical strategy (WATCHMAN left atrial appendage closure device) were similar to the newer pharmacological (novel oral anticoagulants) and dose-adjusted vitamin K antagonists, likely due to a smaller number of patients in the device trials and possibly due to use of dose-adjusted vitamin K antagonists in the device arm being limited to the first two months after WATCHMAN left atrial appendage closure device implantation.

Overall, our findings align with those reported in previously published network meta-analyses [11–16]. However, our analyses also add numerous important findings to the literature. Firstly, we included all stroke prophylaxis strategies (most notably, WATCHMAN left atrial appendage closure devices) and compared them by rankograms in terms of efficacy and safety. Our cluster-ranking analysis has helped to identify the most efficacious and safe strategies. This is of clinical utility as there are multiple FDA approved stroke prophylaxis strategies in patients with non-valvular atrial fibrillation. We also present detailed evidence networks and data derived from FDA Public Summary Documents [31]. Our network meta-analyses also adhere to the most recent PRISMA and GRADE reporting standards **(S2 and S6 Tables)**.

Currently, the stroke prophylaxis guidelines [32] and literature lack the evidence to compare the stroke prophylaxis strategies in terms of efficacy and safety. With the “trade-off” analysis, we have shown that the six available treatments (four novel oral anticoagulants, WATCHMAN left atrial appendage closure devices, and dose-adjusted vitamin K antagonists) can be grouped into three distinct clusters **(Fig 5)**. These three clusters are based on the probabilities of being the most efficacious and safe for patients with non-valvular atrial fibrillation from available randomized controlled trials data to date. Our analysis also highlights the need to rank these strategies from an efficacy and safety standpoint with direct randomized comparisons in future.

There are several limitations with the analysis we employed. Drawbacks of meta-analyses, in general, are well known. The additional limitations of network meta-analyses may include inconsistency and the complex geometry of networks [33]. We did not observe any statistical inconsistencies and the geometry of our network of evidence was not complex. Nonetheless, we emphasize that current clinical decision-making for stroke prophylaxis in patients with non-valvular atrial fibrillation is often based on incomplete evidence, and large head-to-head randomized trials comparing pharmacology (novel oral anticoagulants) and anatomical (WATCHMAN left atrial appendage closure devices) strategies do not exist and are not foreseeable in the near future. Secondly, there is notable heterogeneity in the patients and the study characteristics among the included randomized controlled trials in our analysis. However, the small number of randomized controlled trials that were eligible for inclusion limits any additional analyses that can be conducted to account for heterogeneity in the absence of patient-level data. Thirdly, the randomized controlled trials comparing WATCHMAN left atrial appendage closure devices to dose-adjusted vitamin K antagonists are very small relative to randomized controlled trials comparing novel oral anticoagulants to dose-adjusted vitamin K antagonists. We observed heterogeneity between the two randomized controlled trials comparing this approach. Consequently, we reported random effect estimates in our analysis, which may be more conservative. We also note that the left atrial appendage closure strategy in the WATCHMAN trials required use of dual anti-platelet therapy for at least six months after device implantation. We acknowledge that it is difficult to tease out the influence of dual anti-platelet therapy use in WATCHMAN arm on our results, given the lack of a landmark analysis. Finally, despite the use of robust hierarchical clusters and rankograms, our results should not be used to draw causal or mechanistic inferences given the indirect nature of several comparisons. Clinical judgment should prevail in selecting the most beneficial strategy as well as therapy.

## Conclusions

Novel oral anticoagulants and anatomical strategies (WATCHMAN left atrial appendage closure devices) are all equally effective for ischemic stroke prevention when compared to dose-adjusted vitamin K antagonists but with different safety profiles. The trade-off between safety and efficacy should be the driving force while individualizing strategy as well as choice of therapy in non-valvular atrial fibrillation patients. Hierarchical ranking of the available treatments, as presented in our analysis, can serve as a useful clinical tool to guide selection of therapy in patients with non-valvular atrial fibrillation when deciding between dose-adjusted vitamin K antagonists, novel oral anticoagulants and the WATCHMAN left atrial appendage closure device.

## Supporting Information

S1 Fig Network Forest Plot for Ischemic Stroke. (DOCX)

S2 Fig Network Forest Plot for Major Bleeding. (DOCX)

S3 Fig Network Forest Plot for Primary Safety Endpoint. (DOCX)

S4 Fig Network Funnel Plot. (DOCX)

S1 Section Search Strategy. (DOCX)

S1 Table Definitions. (DOCX)

S2 Table PRISMA Checklist for Network Meta-Analyses. (DOCX)

S3 Table League Table for Log Odds Ratio for Ischemic Stroke Comparisons Estimated by Consistency Modeling (DOCX)

S4 Table League Table for Log Odds Ratio for Major Bleeding Comparisons Estimated by Consistency Modeling. (DOCX)

S5 Table League Table for Log Odds Ratio for Primary Safety Endpoint Comparisons Estimated by Consistency Modeling. (DOCX)

S6 Table Quality Assessment of Comparisons in Accordance with GRADE Guidelines. (DOCX)
