# Supplementary figures and images for: Comparison of Approaches for Stroke Prophylaxis in Patients with Non-Valvular Atrial Fibrillation: Network Meta-Analyses of Randomized Controlled Trials
Source: PLoS One. 2016 Oct 5;11(10):e0163608. doi: 10.1371/journal.pone.0163608 (PMC5051881; doi:10.1371/journal.pone.0163608)

**S1 Figure: Network Forest Plot for Ischemic Stroke**

**
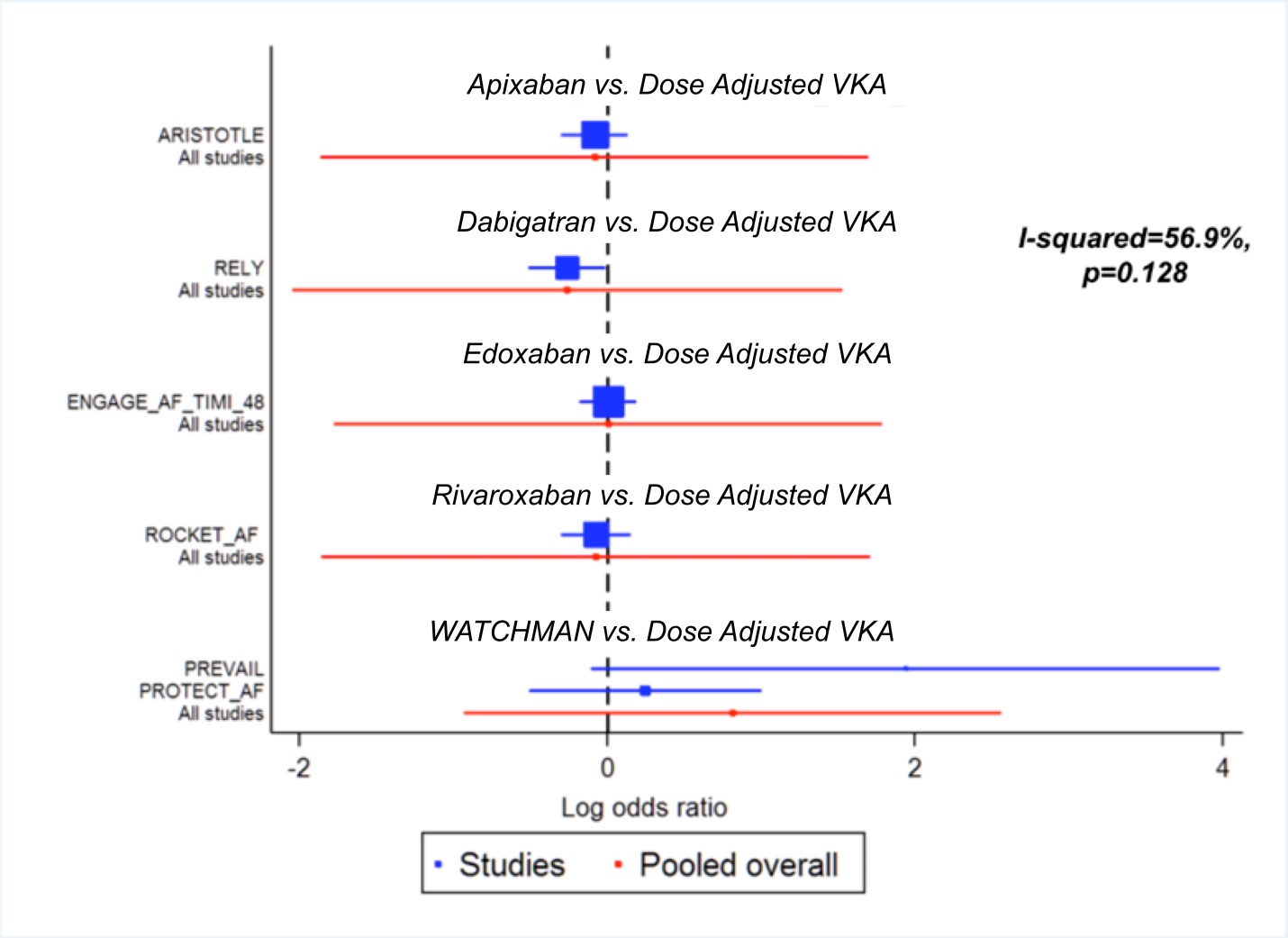
**

VKA = Vitamin K antagonists

Supplement: S1 Fig — (DOCX) [file pone.0163608.s001.docx]

**S2 Figure: Network Forest Plot for Major Bleeding**

**
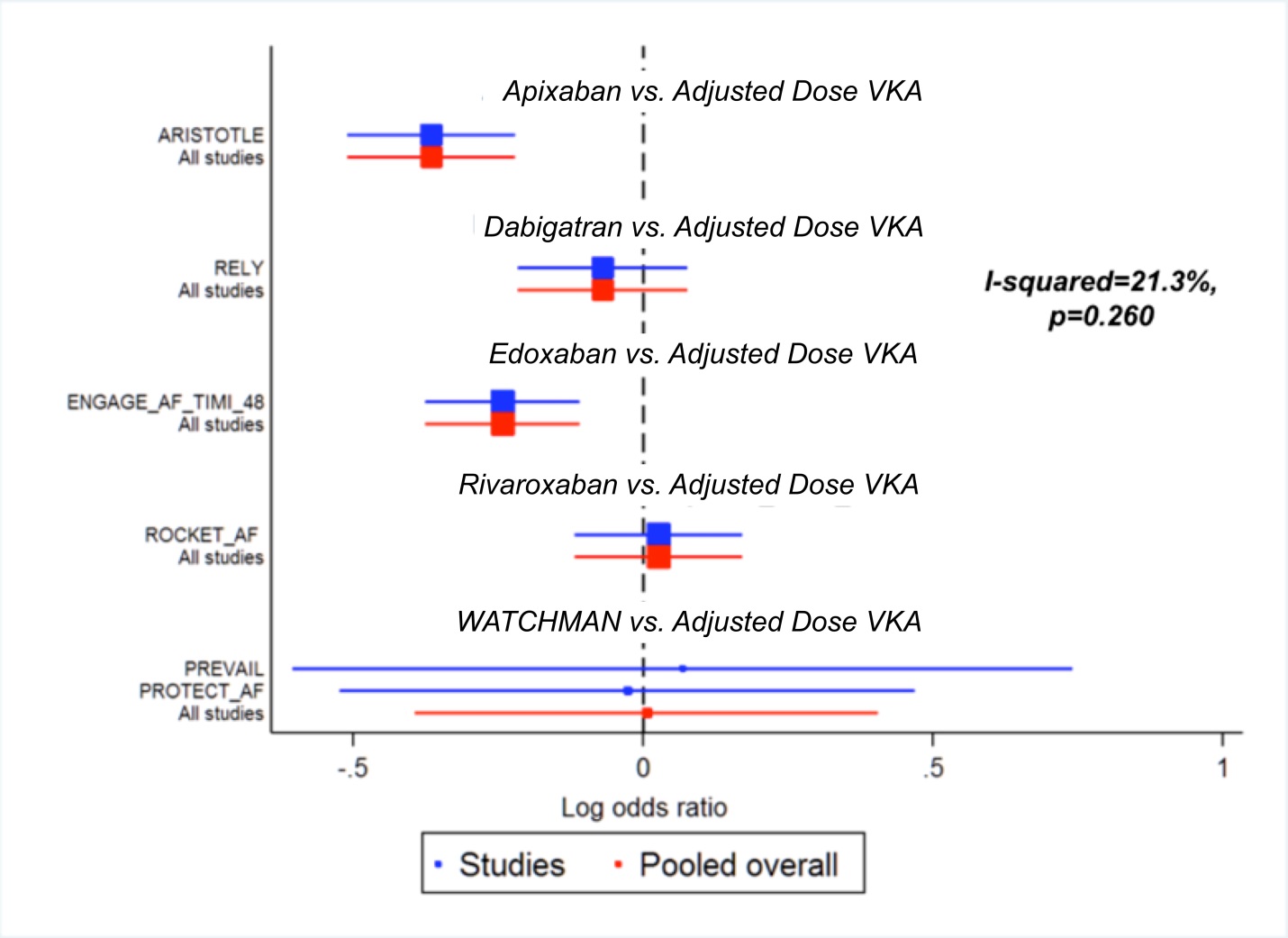
**

VKA = Vitamin K antagonists

Supplement: S2 Fig — (DOCX) [file pone.0163608.s002.docx]

**S3 Figure: Network Forest Plot for Primary Safety Endpoint**


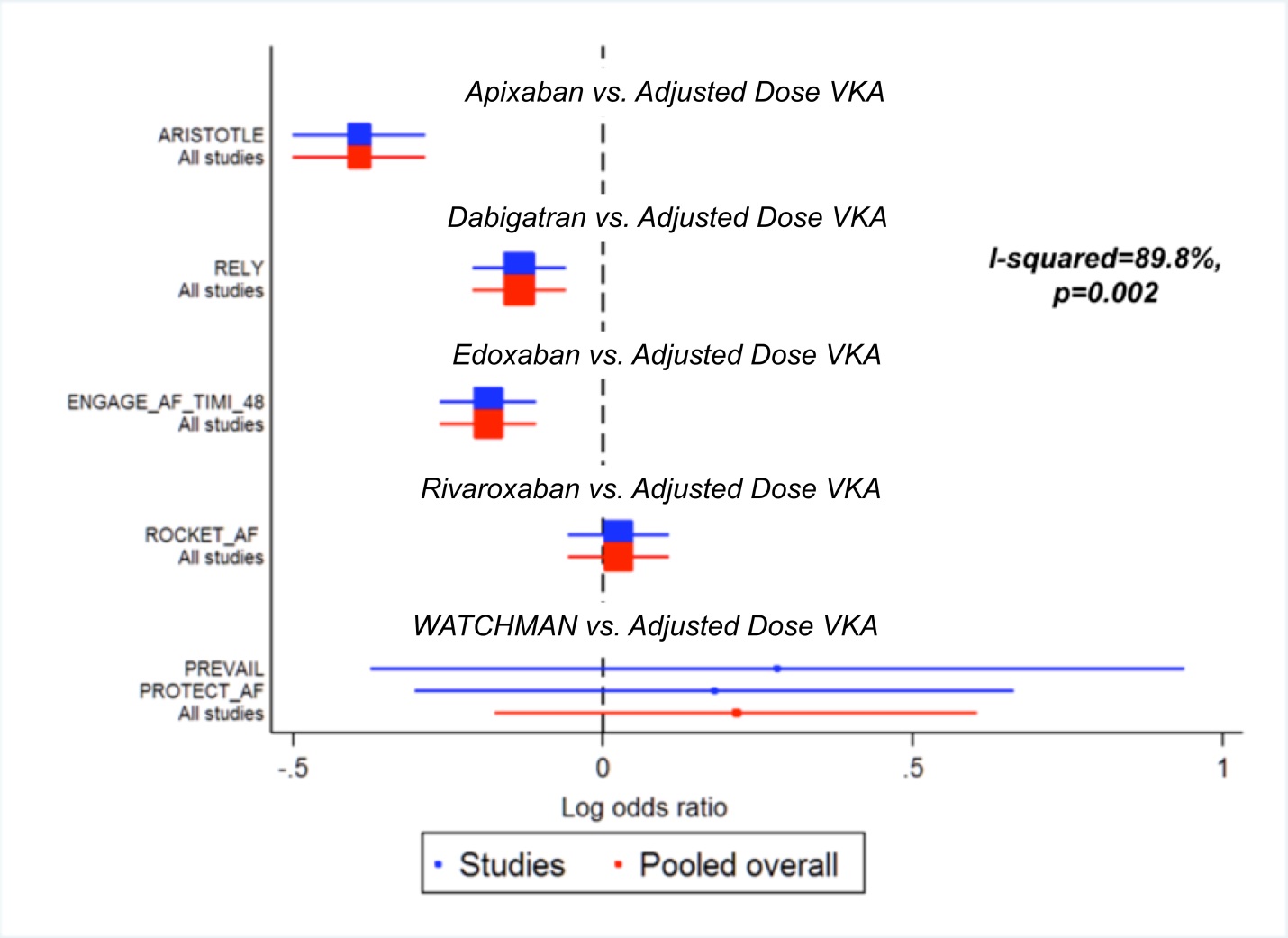
VKA = Vitamin K antagonists

Supplement: S3 Fig — (DOCX) [file pone.0163608.s003.docx]

**S4 Figure: Network Funnel Plot**

**
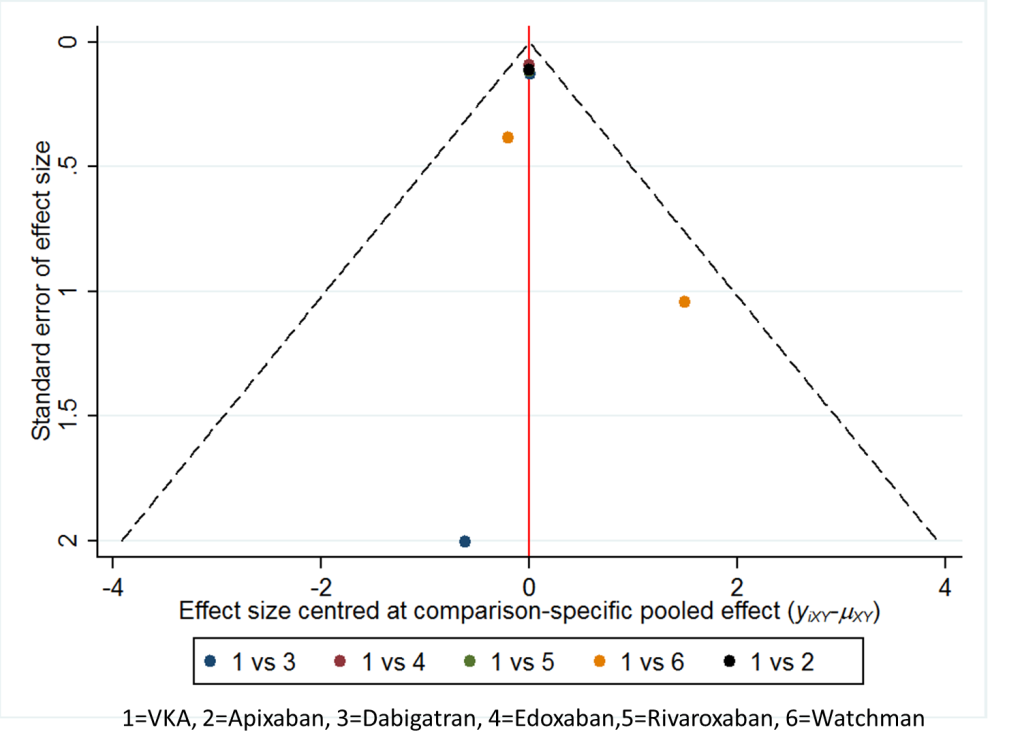
**

VKA = Dose-adjusted Vitamin K antagonists

Supplement: S4 Fig — (DOCX) [file pone.0163608.s004.docx]
