# Supplementary material for: Comparison of Approaches for Stroke Prophylaxis in Patients with Non-Valvular Atrial Fibrillation: Network Meta-Analyses of Randomized Controlled Trials
Source: PLoS One. 2016 Oct 5;11(10):e0163608. doi: 10.1371/journal.pone.0163608 (PMC5051881; doi:10.1371/journal.pone.0163608)
Supplement: S1 Section — (DOCX) [file pone.0163608.s005.docx]

**S1 Section: Search Strategy**

Systematic literature search

An electronic search of SCOPUS will be performed for relevant randomized clinical trials on 10/02/2015. References of identified studies will also manually searched for relevant publications. Three authors (P.A., T.H. and T.T.W.) will independently perform an electronic literature search in SCOPUS using a predefined keywords list. A fourth investigator (N.S.B.) will verify the search results. The following search strategy will be employed:

[http://www.scopus.com/results/results.url?numberOfFields=7&src=s&clickedLink=&edit=t&editSaveSearch=&origin=searchbasic&authorTab=&affiliationTab=&advancedTab=&scint=1&menu=search&tablin=&searchterm1=Atrial+Fibrillation&field1=TITLE_ABS_KEY&connector=AND&searchterm2=Stroke&field2=TITLE_ABS_KEY&connectors=AND&searchTerms=randomized+control+trial&fields=TITLE_ABS_KEY&resetvar=1&resetFormLink=&dateType=Publication_Date_Type&yearFrom=Before+1960&yearTo=Present&loadDate=7&documenttype=All&subjects=LFSC&subjects=HLSC&subjects=PHSC&subjects=SOSC&src=s&st1=Atrial+Fibrillation&st2=Stroke&sot=b&sdt=b&sl=&s=%28TITLE-ABS-KEY%28Atrial+Fibrillation%29+AND+TITLE-ABS-KEY%28Stroke%29AND+TITLE-ABS-KEY%28randomized+control+trial%29%29&sid=73C0806C10C0F0382B8F36430B960AA9.kqQeWtawXauCyC8ghhRGJg%3A350&searchId=73C0806C10C0F0382B8F36430B960AA9.kqQeWtawXauCyC8ghhRGJg%3A350&txGid=73C0806C10C0F0382B8F36430B960AA9.kqQeWtawXauCyC8ghhRGJg%3A35&sort=plf-f&originationType=b&rr](https://webmail.uabmc.edu/owa/redir.aspx?SURL=tN6eEWNg14tSQdQ1I3OfDxNUzhcuLgyUBWz-JZh534x-GsQA8fXSCGgAdAB0AHAAOgAvAC8AdwB3AHcALgBzAGMAbwBwAHUAcwAuAGMAbwBtAC8AcgBlAHMAdQBsAHQAcwAvAHIAZQBzAHUAbAB0AHMALgB1AHIAbAA_AG4AdQBtAGIAZQByAE8AZgBGAGkAZQBsAGQAcwA9ADcAJgBzAHIAYwA9AHMAJgBjAGwAaQBjAGsAZQBkAEwAaQBuAGsAPQAmAGUAZABpAHQAPQB0ACYAZQBkAGkAdABTAGEAdgBlAFMAZQBhAHIAYwBoAD0AJgBvAHIAaQBnAGkAbgA9AHMAZQBhAHIAYwBoAGIAYQBzAGkAYwAmAGEAdQB0AGgAbwByAFQAYQBiAD0AJgBhAGYAZgBpAGwAaQBhAHQAaQBvAG4AVABhAGIAPQAmAGEAZAB2AGEAbgBjAGUAZABUAGEAYgA9ACYAcwBjAGkAbgB0AD0AMQAmAG0AZQBuAHUAPQBzAGUAYQByAGMAaAAmAHQAYQBiAGwAaQBuAD0AJgBzAGUAYQByAGMAaAB0AGUAcgBtADEAPQBBAHQAcgBpAGEAbAArAEYAaQBiAHIAaQBsAGwAYQB0AGkAbwBuACYAZgBpAGUAbABkADEAPQBUAEkAVABMAEUAXwBBAEIAUwBfAEsARQBZACYAYwBvAG4AbgBlAGMAdABvAHIAPQBBAE4ARAAmAHMAZQBhAHIAYwBoAHQAZQByAG0AMgA9AFMAdAByAG8AawBlACYAZgBpAGUAbABkADIAPQBUAEkAVABMAEUAXwBBAEIAUwBfAEsARQBZACYAYwBvAG4AbgBlAGMAdABvAHIAcwA9AEEATgBEACYAcwBlAGEAcgBjAGgAVABlAHIAbQBzAD0AcgBhAG4AZABvAG0AaQB6AGUAZAArAGMAbwBuAHQAcgBvAGwAKwB0AHIAaQBhAGwAJgBmAGkAZQBsAGQAcwA9AFQASQBUAEwARQBfAEEAQgBTAF8ASwBFAFkAJgByAGUAcwBlAHQAdgBhAHIAPQAxACYAcgBlAHMAZQB0AEYAbwByAG0ATABpAG4AawA9ACYAZABhAHQAZQBUAHkAcABlAD0AUAB1AGIAbABpAGMAYQB0AGkAbwBuAF8ARABhAHQAZQBfAFQAeQBwAGUAJgB5AGUAYQByAEYAcgBvAG0APQBCAGUAZgBvAHIAZQArADEAOQA2ADAAJgB5AGUAYQByAFQAbwA9AFAAcgBlAHMAZQBuAHQAJgBsAG8AYQBkAEQAYQB0AGUAPQA3ACYAZABvAGMAdQBtAGUAbgB0AHQAeQBwAGUAPQBBAGwAbAAmAHMAdQBiAGoAZQBjAHQAcwA9AEwARgBTAEMAJgBzAHUAYgBqAGUAYwB0AHMAPQBIAEwAUwBDACYAcwB1AGIAagBlAGMAdABzAD0AUABIAFMAQwAmAHMAdQBiAGoAZQBjAHQAcwA9AFMATwBTAEMAJgBzAHIAYwA9AHMAJgBzAHQAMQA9AEEAdAByAGkAYQBsACsARgBpAGIAcgBpAGwAbABhAHQAaQBvAG4AJgBzAHQAMgA9AFMAdAByAG8AawBlACYAcwBvAHQAPQBiACYAcwBkAHQAPQBiACYAcwBsAD0AJgBzAD0AJQAyADgAVABJAFQATABFAC0AQQBCAFMALQBLAEUAWQAlADIAOABBAHQAcgBpAGEAbAArAEYAaQBiAHIAaQBsAGwAYQB0AGkAbwBuACUAMgA5ACsAQQBOAEQAKwBUAEkAVABMAEUALQBBAEIAUwAtAEsARQBZACUAMgA4AFMAdAByAG8AawBlACUAMgA5AEEATgBEACsAVABJAFQATABFAC0AQQBCAFMALQBLAEUAWQAlADIAOAByAGEAbgBkAG8AbQBpAHoAZQBkACsAYwBvAG4AdAByAG8AbAArAHQAcgBpAGEAbAAlADIAOQAlADIAOQAmAHMAaQBkAD0ANwAzAEMAMAA4ADAANgBDADEAMABDADAARgAwAD)=
