# Supplementary material for: Comparison of Approaches for Stroke Prophylaxis in Patients with Non-Valvular Atrial Fibrillation: Network Meta-Analyses of Randomized Controlled Trials
Source: PLoS One. 2016 Oct 5;11(10):e0163608. doi: 10.1371/journal.pone.0163608 (PMC5051881; doi:10.1371/journal.pone.0163608)
Supplement: S1 Table — (DOCX) [file pone.0163608.s006.docx]

**S1 Table: Definitions**

Where possible, the authors’ definition was used to define the below endpoints. Where the authors’ definition was unclear, we used the below definitions to define our endpoints.

| Stroke | We used the definition outlined by the individual trial’s authors. Persistent clinical neurologic deficit of vascular origin persisting beyond 24 hours. This included hemorrhagic and ischemic strokes that were atraumatic in origin. There were varying requirements for confirmation of occurrence by computed tomography (CT) or magnetic resonance imaging (MRI). Hemorrhagic transformation was excluded from this category, as this was included in the ‘major bleeding’ category. |
| --- | --- |
| Primary safety endpoint | Defined as composite of major bleeding and clinically relevant non-major bleeding in NOAC trials and a composite of major bleeding and any device-related complications in left atrial appendage closure device trials.  Data from FDA documents for left atrial appendage closure device trials were utilized to calculate these numbers |
| Major bleeding | Defined as any bleeding requiring transfusion of more than two units of packed red blood cells, any visceral bleeding leading to hemodynamic instability, any bleeding requirement operative intervention, or any fatal bleeding. Data from FDA documents for left atrial appendage closure device trials were utilized to calculate these numbers |
| Non-major clinically relevant bleeding | All bleeding events reported by authors that were not classified as ‘major bleeding’ events. |
