# Supplementary material for: Comparison of Approaches for Stroke Prophylaxis in Patients with Non-Valvular Atrial Fibrillation: Network Meta-Analyses of Randomized Controlled Trials
Source: PLoS One. 2016 Oct 5;11(10):e0163608. doi: 10.1371/journal.pone.0163608 (PMC5051881; doi:10.1371/journal.pone.0163608)
Supplement: S3 Table — (DOCX) [file pone.0163608.s008.docx]

**S3 Table: League Table for Odds Ratio for Ischemic Stroke Comparisons Estimated by Consistency Modeling**

| **WATCHMAN** | **VKA** | **Rivaroxaban** | **Edoxaban** | **Dabigatran** | **Apixaban** |
| --- | --- | --- | --- | --- | --- |
| **WATCHMAN** | 0.44 (0.08,2.54) | 0.41 (0.03,4.98) | 0.44 (0.04,5.38) | 0.34 (0.03,4.15) | 0.41 (0.03,4.94) |
| 2.26 (0.39,12.96) | **VKA** | 0.93 (0.16,5.52) | 1.00 (0.17,5.96) | 0.77 (0.13,4.61) | 0.92 (0.15,5.48) |
| 2.44 (0.20,29.62) | 1.08 (0.18,6.43) | **Rivaroxaban** | 1.08 (0.09,13.48) | 0.83 (0.07,10.40) | 0.99 (0.08,12.38) |
| 2.25 (0.19,27.23) | 1.00 (0.17,5.90) | 0.92 (0.07,11.47) | **Edoxaban** | 0.77 (0.06,9.56) | 0.92 (0.07,11.38) |
| 2.93 (0.24,35.71) | 1.30 (0.22,7.76) | 1.20 (0.10,15.04) | 1.30 (0.10,16.25) | **Dabigatran** | 1.19 (0.10,14.92) |
| 2.46 (0.20,29.80) | 1.09 (0.18,6.46) | 1.01 (0.08,12.55) | 1.09 (0.09,13.56) | 0.84 (0.07,10.46) | **Apixaban** |

VKA = Vitamin K antagonists
