# Supplementary material for: Comparison of Approaches for Stroke Prophylaxis in Patients with Non-Valvular Atrial Fibrillation: Network Meta-Analyses of Randomized Controlled Trials
Source: PLoS One. 2016 Oct 5;11(10):e0163608. doi: 10.1371/journal.pone.0163608 (PMC5051881; doi:10.1371/journal.pone.0163608)
Supplement: S4 Table — (DOCX) [file pone.0163608.s009.docx]

**S4 Table League Table for Odds Ratio for Major Bleeding Comparisons Estimated by Consistency Modeling**

| **WATCHMAN** | **VKA** | **Rivaroxaban** | **Edoxaban** | **Dabigatran** | **Apixaban** |
| --- | --- | --- | --- | --- | --- |
| **WATCHMAN** | 0.99 (0.67,1.48) | 1.02 (0.67,1.56) | 0.78 (0.51,1.19) | 0.93 (0.61,1.42) | 0.69 (0.45,1.05) |
| 1.01 (0.67,1.50) | **VKA** | 1.03 (0.89,1.19) | 0.78 (0.69,0.90) | 0.93 (0.81,1.08) | 0.69 (0.60,0.80) |
| 0.98 (0.64,1.50) | 0.97 (0.84,1.13) | **Rivaroxaban** | 0.76 (0.63,0.93) | 0.91 (0.74,1.11) | 0.68 (0.55,0.83) |
| 1.28 (0.84,1.95) | 1.27 (1.12,1.46) | 1.31 (1.08,1.59) | **Edoxaban** | 1.19 (0.97,1.45) | 0.88 (0.73,1.08) |
| 1.08 (0.71,1.65) | 1.07 (0.93,1.24) | 1.10 (0.90,1.35) | 0.84 (0.69,1.03) | **Dabigatran** | 0.74 (0.61,0.91) |
| 1.45 (0.95,2.22) | 1.44 (1.25,1.67) | 1.48 (1.21,1.82) | 1.13 (0.93,1.38) | 1.34 (1.09,1.65) | **Apixaban** |

VKA = Vitamin K antagonists
