# Supplementary material for: Comparison of Approaches for Stroke Prophylaxis in Patients with Non-Valvular Atrial Fibrillation: Network Meta-Analyses of Randomized Controlled Trials
Source: PLoS One. 2016 Oct 5;11(10):e0163608. doi: 10.1371/journal.pone.0163608 (PMC5051881; doi:10.1371/journal.pone.0163608)
Supplement: S5 Table — (DOCX) [file pone.0163608.s010.docx]

**S5 Table: League Table for Odds Ratio for Primary Safety Endpoint Comparisons Estimated by Consistency Modeling**

| **WATCHMAN** | **VKA** | **Rivaroxaban** | **Edoxaban** | **Dabigatran** | **Apixaban** |
| --- | --- | --- | --- | --- | --- |
| **WATCHMAN** | 0.82 (0.55,1.20) | 0.84 (0.56,1.25) | 0.68 (0.46,1.01) | 0.71 (0.48,1.06) | 0.55 (0.37,0.82) |
| 1.23 (0.83,1.81) | **VKA** | 1.03 (0.95,1.11) | 0.83 (0.77,0.90) | 0.88 (0.81,0.94) | 0.67 (0.61,0.75) |
| 1.20 (0.80,1.78) | 0.98 (0.90,1.06) | **Rivaroxaban** | 0.81 (0.72,0.91) | 0.85 (0.76,0.95) | 0.66 (0.57,0.75) |
| 1.48 (0.99,2.20) | 1.20 (1.11,1.30) | 1.23 (1.10,1.38) | **Edoxaban** | 1.05 (0.95,1.17) | 0.81 (0.71,0.93) |
| 1.40 (0.94,2.08) | 1.14 (1.06,1.23) | 1.17 (1.05,1.31) | 0.95 (0.85,1.06) | **Dabigatran** | 0.77 (0.68,0.88) |
| 1.82 (1.21,2.72) | 1.48 (1.33,1.65) | 1.52 (1.33,1.74) | 1.23 (1.08,1.41) | 1.30 (1.14,1.48) | **Apixaban** |

VKA = Vitamin K antagonists
