# Supplementary material for: Comparison of Approaches for Stroke Prophylaxis in Patients with Non-Valvular Atrial Fibrillation: Network Meta-Analyses of Randomized Controlled Trials
Source: PLoS One. 2016 Oct 5;11(10):e0163608. doi: 10.1371/journal.pone.0163608 (PMC5051881; doi:10.1371/journal.pone.0163608)
Supplement: S6 Table — (DOCX) [file pone.0163608.s011.docx]

**S6 Table: Quality Assessment of Comparisons in Accordance with GRADE** **Guidelines**

| Comparison | Direct Evidence Estimates | | | Indirect Evidence Estimates | | | Network Meta-Analysis Estimates | | |
| --- | --- | --- | --- | --- | --- | --- | --- | --- | --- |
|  | Quality of Evidence | | | Quality of Evidence | | | Quality of Evidence | | |
|  | Ischemic Stroke | Major Bleeding | Primary Safety Endpoint | Ischemic Stroke | Major Bleeding | Primary Safety Endpoint | Ischemic Stroke | Major Bleeding | Primary Safety Endpoint |
| WATCHMAN vs. VKA | +++ | +++ | +++ | ++ | ++ | ++ | ++ | ++ | ++ |
| WATCHMAN vs. Apixaban | _ | _ | _ | ++ | ++ | ++ | ++ | ++ | ++ |
| WATCHMAN vs. Dabigatran | _ | _ | _ | ++ | ++ | ++ | ++ | ++ | ++ |
| WATCHMAN vs. Rivaroxaban | _ | _ | _ | ++ | ++ | ++ | ++ | ++ | ++ |
| WATCHMAN vs. Edoxaban | _ | _ | _ | ++ | ++ | ++ | ++ | ++ | ++ |
| Apixaban vs. VKA | ++++ | ++++ | ++++ | ++ | ++ | ++ | ++ | ++ | ++ |
| Apixaban vs. Dabigatran | _ | _ | _ | ++ | ++ | ++ | ++ | ++ | ++ |
| Apixaban vs. Rivaroxaban | _ | _ | - | ++ | ++ | ++ | ++ | ++ | ++ |
| Apixaban vs. Edoxaban | _ | _ | _ | ++ | ++ | ++ | ++ | ++ | ++ |
| Dabigatran vs. VKA | ++++ | ++++ | ++++ | ++ | ++ | ++ | ++ | ++ | ++ |
| Dabigatran vs. Rivaroxaban | _ | _ | _ | ++ | ++ | ++ | ++ | ++ | ++ |
| Dabigatran vs. Edoxaban | _ | _ |  | ++ | ++ | ++ | ++ | ++ | ++ |
| Rivaroxaban vs. VKA | ++++ | ++++ | ++++ | ++ | ++ | ++ | ++ | ++ | ++ |
| Rivaroxaban vs. Edoxaban | _ | _ | _ | ++ | ++ | ++ | ++ | ++ | ++ |
| Edoxaban vs. VKA | ++++ | ++++ | ++++ | ++ | ++ | ++ | ++ | ++ | ++ |

+ Very low quality, ++ Low quality, +++ Moderate quality, ++++ High quality
